# Ceria nanoparticles alleviate myocardial ischemia-reperfusion injury by inhibiting cardiomyocyte apoptosis via alleviating ROS mediated excessive mitochondrial fission

**DOI:** 10.1016/j.mtbio.2025.101770

**Published:** 2025-04-17

**Authors:** Ying Sun, Jiabao Xu, Ling Zou, Yan Tan, Jie Li, Haoran Xin, Yanli Guo, Weikai Kong, Dingyuan Tian, Xinyu Bao, Xiaoqin Wan, Xiaoxu Li, Zhihui Zhang, Xiaochao Yang, Fang Deng

**Affiliations:** a Department of Pathophysiology, College of High Altitude Military Medicine, Army Medical University, Chongqing, 400038, China; b Department of Cardiovascular Medicine, Center for Circadian Metabolism and Cardiovascular Disease, Southwest Hospital, Army Medical University, Chongqing, 400038, China; c School of Biomedical Engineering and Medical Imaging, Army Medical University, Chongqing, 400038, China; d Key Laboratory of Extreme Environmental Medicine, Ministry of Education of China, Chongqing, 400038, China; e Key Laboratory of High Altitude Medicine, PLA, Chongqing, 400038, China; f Key Laboratory of Geriatric Cardiovascular and Cerebrovascular Disease, Ministry of Education of China, Chongqing, 400038, China; g Department of Ultrasound, Southwest Hospital, Army Medical University, Chongqing, 400038, China; h Institute of Pathology and Southwest Cancer Center, Southwest Hospital, Army Medical University, Chongqing, 400038, China

**Keywords:** Myocardial ischemia-reperfusion injury, Ceria nanoparticles, Reactive oxygen species, Oxidative stress, Mitochondria

## Abstract

Reperfusion through thrombolytic therapy or primary percutaneous coronary intervention is commonly used to deal with acute myocardial infarction. However, the reperfusion procedure is accompanied by myocardial ischemia-reperfusion injury (MIRI). Currently, there is no therapeutics that can effectively deal with MIRI in clinical practice. Herein, the potential of ceria nanoparticles (CNPs) coated by different ligands in the treatment of rat MIRI is evaluated. The results demonstrate that CNPs can effectively modulate the oxidative stress in the heart tissue through the elimination of reactive oxygen species (ROS) and stimulation of endogenous antioxidant system. The inhibition of oxidative stress results in the reduction of p-Drp1 (Ser 616) which is critical in driving the fission and fragmentation of mitochondria. The improved mitochondrial dynamics saves the cardiomyocytes from apoptosis and reduces the acute injury of left ventricular wall during the MIRI. The ejection function of the left ventricle for both the short-term and long-term MIRI rats is well preserved. We therefore believe based on these results that the administration of CNPs is beneficial in the attenuation of MIRI during the acute stage. These findings provide useful information for the future fabrication of inorganic antioxidant nanomedicine for the treatment of MIRI.

## Graphical abstract

**Figure:**
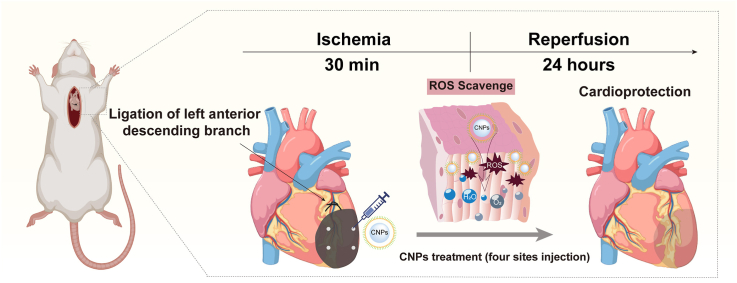

## 1 Introduction

Acute myocardial infarction is an important cause of death worldwide [1]. Reperfusion through thrombolytic therapy or primary percutaneous coronary intervention is commonly used to deal with this condition [2,3]. However, ischemia and reperfusion can induce myocardial damage, a phenomenon known as myocardial ischemia-reperfusion injury (MIRI), which can cause important complications such as cardiac arrhythmia and heart failure [4]. Strategies including leucocyte therapy, ischemic preconditioning, antioxidant therapy, various pharmacological options, and complement therapy have been used to ameliorate MIRI [[5], [6], [7], [8], [9], [10], [11], [12]]. Although promising preclinical results have been reported, the translation of these strategies into human clinical trials was disappointing [13]. From this perspective, the treatment of MIRI remains a great challenge in clinical practice.

The urgency of exploring new therapies to deal with MIRI have driven intensive studies on the molecular and cellular mechanisms underlying the occurrence and development of this condition. Accordingly, many pathways including mitochondrial dysfunction, metabolic alterations, inflammation, oxidative stress and autophagy deregulation have been considered as the important reasons for MIRI [[14], [15], [16], [17], [18]]. Within these, the mitochondrial pathway plays a critical role in the development of MIRI. Mitochondria account for approximately 20 %–35 % volume of the cardiomyocytes. They drive the contraction of the cardiomyocytes through the generation of adenosine triphosphate and thus are the energy centers of the cardiomyocytes. Fission and fusion are two important ways for mitochondria to maintain their homeostasis and function [19]. However, during the ischemia and reperfusion, the over accumulation of reactive oxygen species (ROS) can stimulate excessive mitochondrial fission resulting in the generation of large amounts of mitochondrial fragments [20]. The fragmentation of mitochondria in turn can induce the generation of additional ROS and thus a vicious cycle is formed. The presence of excessive mitochondrial fragments and ROS can seriously threaten the survival of the cardiomyocytes. On the other hand, previous studies have proved that the mitochondrial fission and fragmentation are determined by the translocation of the mitochondrial fission protein dynamin-related protein 1 (Drp1) onto the mitochondrial membranes [21]. The phosphorylation of Drp1 (p-Drp1) at the mitochondrial membranes including p-Drp1 (Ser 616) and p-Drp1 (Ser 637), can directly drive the fission and fragmentation of mitochondria. Among them, p-Drp1 (Ser 616) promotes mitochondrial fission, but p-Drp1 (Ser 637) inhibits it [22]. Therefore, the regulation of p-Drp1 is a potential way to attenuate the MIRI.

**Scheme 1:**
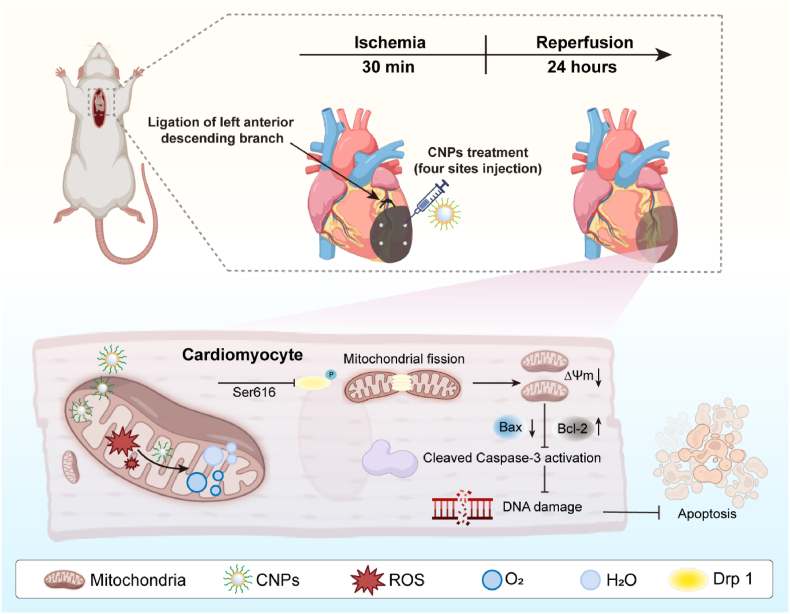
Ceria nanoparticles attenuate MIRI through the inhibition of cardiomyocytes apoptosis.

Recently, the exploration of ceria nanoparticles (CNPs) for biomedical applications have drawn considerable attention. There are two oxidation states (Ce^4+^ and Ce^3+^) for cerium atoms in CNPs crystals, and these two electronic configurations have the ability to easily and drastically exchange depending on the oxygen partial pressure of the surroundings. Based on these properties, they can mimic the catalytic activity of several enzymes, especially superoxide dismutase and catalase which can easily scavenge ROS under physiological conditions [23]. We have recently reported that CNPs could ameliorate renal ischemia-reperfusion injury based on its ability to curb oxidative stress and inflammatory responses [24]. Other studies have shown that CNPs could decrease the hepatic and cerebral ischemia-reperfusion injury [[25], [26], [27]]. These studies implied that the MIRI might benefit from the administration of CNPs. Herein, we evaluated the potential of ∼4 nm sized CNPs coated by different ligands in the treatment of MIRI. Our results indicated that the CNPs could effectively modulate ROS accumulation in the heart tissue within the pathological process of MIRI. The decrease of ROS level inhibited the mitochondrial fission through the Drp1 phosphorylation pathway. The preservation of mitochondrial structure and function in turn resulted in the attenuation of cardiomyocytes apoptosis in the ischemia-reperfusion region (Scheme 1). Finally, in the short-term (24 h) and long-term (4 weeks) animal models, the CNPs exhibited great potential in protecting the heart tissue from MIRI.

## 2 Experimental section

### 2.1 Materials

Cerium (III) nitrate hexahydrate (Ce(NO_3_)_3_·6H_2_O) and 1-octadecebe were purchased from Acros. Trioctylphosphine oxide, succinic acid (SA), polyethylene glycol diacid, oleylamine, (−)-riboflavin, nitrotetrazoliumblue chloride (NBT), and N-hydroxysuccinimide (NHS) were purchased from Sigma-Aldrich. N-(3-Dimethylaminopropyl)-N′-ethylcarbodiimide hydrochloride (EDC) was purchased from Aladdin. DMEM medium, 0.25 % trypsin, penicillin and streptomycin were purchased from GIBOC. Fetal bovine serum (FBS) was purchased from Lonsera. TUNEL, DCFH-DA, T-SOD assay kit and antifade mounting medium with DAPI were purchased from Beyotime. TTC staining solution, bovine serum albumin (BSA), modified sirius red staining solution, masson's trichrome staining kit and DMEM low glucose were purchased from Solarbio. Annexin V-FITC apoptosis detection iit was purchased from BD Bioscience. Cell counting kit-8 (CCK-8) kit was purchased from MedChemExpress. Trizol, Alexa Fluor TM 488 goat anti-rabbit IgG (H + L), Alexa Fluor TM 594 goat anti-mouse IgG (H + L) and Alexa Fluor TM 555 goat anti-rabbit were purchased from Invitrogen. GoScript™ reverse transcription mix, oligo(dT) kit was purchased from Promega. 2 × SP qPCR Mix was purchased from Bioground. Immunohistochemistry (IHC) detection system kit was purchased from Bioss. CK, LDH, CRE, AST, ALT, MDA assay kit and cTn-I) and CK-MB ELISA kits were purchased from Jiancheng. Rabbit anti-Caspase-3, C.Caspase-3, Drp1, p-Drp1 (Ser616), HSP90, Bax and Bcl-2 antibody were purchased from Cell Signaling technology. Goat Anti-Rabbit IgG (H + L) were purchased from PTM Bio. H9c2 and HUVEC cell lines were purchased from ATCC.

### 2.2 Synthesis of CNPs

The CNPs was synthesized by thermal decomposition method according to our previous procedures [28]. Briefly, Ce(NO_3_)_3_·6H_2_O (700 mg) and trioctylphosphine oxide (1g) were dissolved in ethanol (3 mL). The mixture was added 1-octadecene (5 mL) and heated to 90 °C under vacuum conditions for 15 min to remove the ethanol. After that, the mixture was added oleylamine (100 μL) and heated to 190 °C. The reaction was maintained at this temperature for 15 min. After cooling to room temperature, the product was washed by acetone and toluene for several times to remove the byproducts. The CNPs were dispersed in tetrahydrofuran (10 mL) and stored at 4 °C.

### 2.3 Surface modification of CNPs

The CNPs was modified by SA, PEG600 and PEG2000 ligands according to our previous method. In detail, a certain amount of ligand (SA: 109 mg, PEG600: 554 mg, PEG2000: 1847 mg), NHS (105 mg) and EDC (175 mg) were mixed in dichloromethane (10 mL) and activated at room temperature for 8 h. After removing the solvent by rotate evaporation, alendronate (100 mg) and Na_2_CO_3_ (250 mg) dissolved in 20 mL H_2_O were added to the activated ligand. The reaction was carried out at room temperature for 24 h. After that, the synthesized CNPs (1 mL) was diluted by tetrahydrofuran (5 mL) and mixed with the ligand solution (5 mL). The mixture was stirred at 80 °C for 8 h. After cooling to room temperature, the water layer was separated and precipitated out by acetone. The surface modified CNPs were dialyzed in ultrapure water for 24 h.

### 2.4 Characterization of CNPs

The CNPs was characterized by TEM, XRD and TGA. The TEM samples were prepared by dropping the CNPs dispersed in tetrahydrofuran onto ultrathin carbon copper grids, and TEM images were scanned on a Tecnai G2 F20 S-TWIN operated at 200 kV. The XRD graph was measured on a Rigaku MiniFlex600 instrument equipped with a Cu Kα radiation source operated at 40 kV and 15 mA with a scanning speed of 1°/min. The TGA was measured on a TA SDT650 instrument with heating rate of 20 °C/min.

### 2.5 ROS scavenging activity assay

The ROS scavenging activity of the CNPs was tested by our previous method. In detail, a testing solution containing 800 μL EDTA (0.1 M), 300 μL NBT (2 mM), 11.4 mL sodium phosphate buffer (10 mM, pH 7.8) and 200 μL riboflavin (0.6 mM) was prepared before running the assay. The CNPs were diluted by sodium phosphate buffer (10 mM, pH 7.8). For the assay reaction, 100 μL testing solution and 50 μL CNPs were mixed in 96-well plates and shook for 60 s. After that, the solution was illuminated under white light for 90 s. The ROS inhibition ratios were determined by calculating the decrease of absorbance at 560 nm.

### 2.6 Cell viability assay

H9c2 cells and HUVEC cells were cultured in humidified atmosphere (5 % CO_2_) at 37 °C, and grown in DMEM medium supplemented with 10 % FBS and 1 % penicillin/streptomycin. HUVEC was human umbilical vein endothelial cells. H9c2 cells (5000 cells/well) and HUVEC cells (8000 cells/well) were seeded on 96-well plate and grown in DMEM medium supplemented with 10 % FBS and 1 % penicillin/streptomycin for 24 h. The CNPs coated by different ligands were added to the medium and incubated for 24 h. After that, the medium was replaced by fresh medium containing CCK-8 reagent (10: 1) and incubated at 37 °C for 2 h. The absorbance of the medium at 450 nm was detected on a microplate reader (Synergy4, Bio Tek).

### 2.7 Establishment of cell OGD injury model

H9c2 cells (5000 cells/well) were seeded on 96-well plate and grown in DMEM medium supplemented with 10 % FBS and 1 % penicillin/streptomycin for 24 h. To induce the OGD injury, the cells were cultured in a humidified atmosphere at 37 °C in the presence of 5 % CO_2_, 2 % O_2_ and 93 % N_2_, and grown in DMEM medium without serum and glucose for 6 h. After that, the cells were transferred to normal culture condition (37 °C, 5 % CO_2_), and grown in DMEM medium supplemented with 10 % FBS and 1 % penicillin/streptomycin for 24 h in the presence of different concentrations of CNPs (0, 10, 50, 100, 200, 500 μM). The cell viabilities were tested by CCK-8 as described above.

### 2.8 Establishment of MIRI rat model

Male SD rats were purchased from the Army Medical University. All animal surgical operations were performed in accordance with the Guidelines for Care and Use of Laboratory Animals of Army Medical University. Protocols were approved by the Animal Ethics Committee of Army Medical University (AMUWEC20228030). The animals were fasted for 12 h before operation but were allowed free access to water. The animals were anesthetized by the inhalation of isoflurane (1–1.5 %) in O_2_ using an anesthetic equipment and ventilated using a rodent ventilator. The stroke volume and frequency of breath were set by the ventilator. The heart was exposed through a left-sided mini-thoracotomy. To establish the MIRI model, the rat was subjected to left anterior descending coronary artery ligation. For the treatment, CNPs (10 mg/kg) dissolved in saline (100 μL) were injected into the heart near the ligation tissue (four sites, 25 μL/site). After ligation for 30 min, the propene sutures were cut off to allow for reperfusion. The rat model was divided into six groups: sham operation, normal saline, SA, PEG600 and PEG2000.

### 2.9 In vitro and *in vivo* ROS analysis

For the *in vitro* ROS measurement, H9c2 cells (1.5 × 10^5^ cells/well) were seeded on 24-well plate and grown in DMEM medium supplemented with 10 % FBS and 1 % penicillin/streptomycin for 24 h. The procedures for the induction of OGD were the same as described above. After OGD injury, the medium was changed to normal medium containing 200 μM CNPs and grown for 24 h. Then, the cells were treated by DCFH-DA dissolved in DEME medium (1: 1000) for 20 min. The cells were rinsed by serum-free high-glucose medium for three times before taking fluorescent images. For the *in vivo* ROS measurement, the MIRI model was established and the CNPs was administered according to the methods described above. The rats were sacrificed 24 h after CNPs treatment and the hearts were taken out for frozen sections (7 μm). The slices were incubated with serum-free high-glucose medium containing DCFH-DA (1:1000) at 37 °C for 20 min, and washed with pre-cooled PBS for three times. Finally, the slices were mounted by DAPI included Antifade Mounting Medium. Immunofluorescence images were acquired using a whole slide scanner (Olympus Slide VS200).

### 2.10 TTC staining

After reperfusion for 24 h, the rats were euthanized and the hearts were harvested. The hearts were cut into six transverse slices with same thickness on a cutting mold. The slices were incubated with 1 % TTC solution in dark at 37 °C for 10 min. After washing twice with PBS, the images were taken on a scanner (Lide400, Canon). The infarcted myocardium was stained in white and the viable tissue was stained in red. The percentage of the infarct areas were analyzed by Image J software.

### 2.11 Echocardiography (ECG) analysis

The cardiac function was analyzed at day 1 and day 28 after MIRI using transthoracic echocardiography. The rats were anesthetized by the inhalation of isoflurane (1–1.5 %) in O_2_. Their chest hair was completely removed before the test using depilatory paste. M-mode echocardiography was performed on a Vevo 2100 instrument (Visual Sonic, Canada).

### 2.12 Histological analysis

For the hematoxylin and eosin (HE) staining, the heart tissues were fixed in 4 % paraformaldehyde solution for 24 h followed by embedded in paraffin. Tissue sections (5 μm) were used for HE staining. The HE staining images were taken on a photomicroscope (Olympus VS200). For the masson trichrome staining and sirius red staining, the heart tissues were fixed in 4 % paraformaldehyde solution for 24 h and then embedded in paraffin. Tissue sections (5 μm) were used for masson trichrome and sirius red staining. The masson trichrome and sirius red staining images were taken on a photomicroscope (Olympus VS200). For the immunofluorescence staining, the sections were prepared according to procedures provided by the kit manufacturer. The sections were blocked by blocking solution for 20 min. The blocked sections were added first antibody and incubated at 4 °C overnight. After washing by PBS for three times, the sections were added secondary antibody and incubated at room temperature for 30 min. The sections were mounted and the images were taken on a photomicroscope (Olympus VS200). For the TUNEL assay, the sections were dewaxed and added 20 μg/mL DNase-free proteinase K. After incubating at 37 °C for 30 min, the sections were washed by PBS for three times. TUNEL detection solution was added to the sections and incubated at 37 °C for 1 h in the dark. After washing with PBS for three times, the sections were mounted by DAPI contained Antifade Mounting Medium. The images were acquired on a whole slide scanner (Olympus Slide VS200).

### 2.13 Plasma biochemistry analysis

The rat blood samples were collected via the heart 24 h after reperfusion using a vacuum tube. After standing at room temperature for 30 min, the serum was separated by centrifugation at 5000 rpm for 10 min. The creatinine, AST, ALT, T-SOD, MDA, CK, LDH, cTn-I, and CK-MB were detected according to the procedures provided by the manufacturers of the detection kits.

### 2.14 Quantification of the cerium in heart tissue

The rats were administered with CNPs according to the procedure described above. After the injection for 24 h, the heart tissue was collected and digested by nitric acid at 60 °C for 4 h. After dilution, the cerium level was quantified by ICP-AES (PerkinElmer Avio 200).

### 2.15 qRT-PCR analysis

Total RNA was isolated from heart tissue using the procedures provided by the reagent manufacturer. The RNA was reverse transcribed into cDNA using the reverse transcription kit and the expression of the genes was detected on a CFX connect system (Bio-Rad).

### 2.16 Western blot analysis

The proteins (15 μg) extracted from the heart were separated by 12 % SDS-PAGE followed by transferring onto PVDF membranes. After blocking in 5 % skim milk or BSA, membranes were incubated with first antibodies at 4 °C overnight. The membranes were incubated with second antibody for 1 h. After each operation, the strips were rinsed three times (5 min each time) with TBST. Finally, the strips were revealed by a ChemiDoc MP Imaging System (Bio-Rad).

### 2.17 Flow cytometry

The Annexin V-FITC apoptosis detection kit was selected to detect the apoptosis of H9c2 cells. The procedures for the induction of OGD injury were the same as described above. After OGD injury for 4 h, the cells were treated by CNPs and rinsed by pre-warmed PBS. The cells (10 × 10^4^ cells) were suspended in Annexin Ⅴ binding buffer (195 μL/tube). After that, Annexin Ⅴ-FITC (5 μL/tube) and PI (10 μL/tube) were added to the cells and incubated at room temperature for 20 min in the dark. The samples were tested on a CytoFLEX instrument (BECKMAN COULTER Life Science).

### 2.18 Statistical analysis

Statistical analysis was performed on GraphpadPrism7.0 software. The data was presented as presented as mean ± standard error of the mean (SEM). Statistical evaluations of data were performed using the one-way analysis of variance (ANOVA) and Bonferroni correction. The levels of statistically significant were set as ∗*p* < 0.05, ∗∗*p* < 0.01, ∗∗∗*p* < 0.001, ∗∗∗∗*p* < 0.0001.

## 3 Results and discussion

### 3.1 Synthesis and biocompatibility of CNPs

**Fig. 1:**
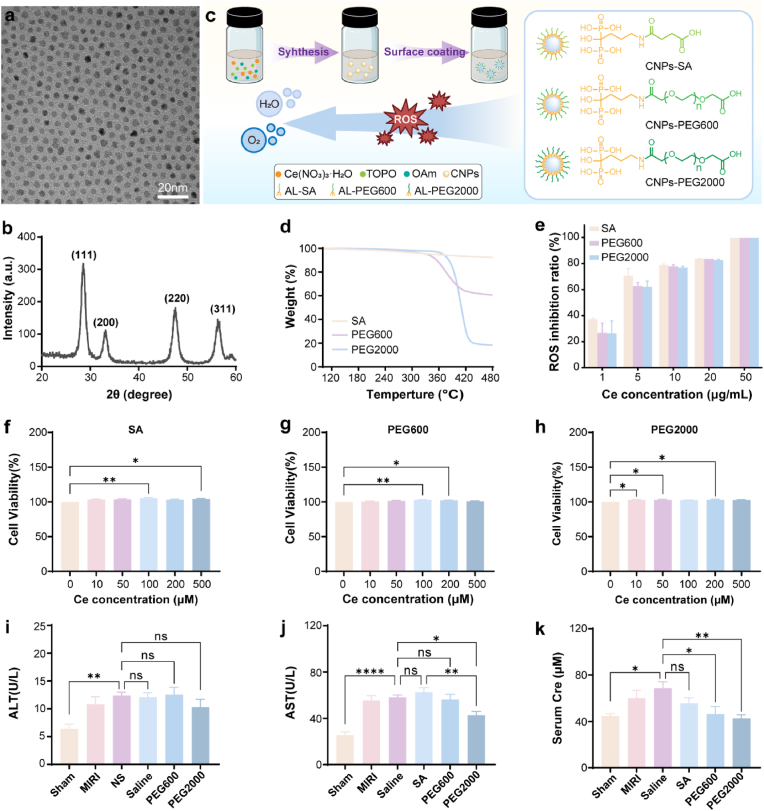
Synthesis and biocompatibility of CNPs. TEM image (a) and XRD spectrum (b) of the synthesized CNPs. (c) Scheme of the surface modification of CNPs with different coating molecules. (d) TGA graphs of the surface-coated CNPs. (e) ROS inhibition ratios of the surface-coated CNPs tested by superoxide dismutase mimetic activity assay. (f–h) Cell viability of the surface coated CNPs measured by CCK-8 (n = 6). (i–k) Serum ALT, AST and Cre levels of the rats 24 h after the injection of CNPs (n = 6). Data were presented as mean ± SEM. ∗*p* < 0.05, ∗∗*p* < 0.01, ∗∗∗*p* < 0.001, ∗∗∗∗*p* < 0.0001, ns not significant.

The CNPs featured with size of ∼4 nm were used in this study because the smaller-sized nanoparticles could better diffuse within the heart tissue than the bigger-sized nanoparticles. The morphology of the synthesized CNPs was characterized by transmission electron microscope (TEM). The CNPs demonstrated near spherical shape and their size distribution was narrow (Fig. 1a). The ceria nature of the crystal was confirmed by X-ray diffraction (XRD) as the typical ceria cubic fluorite peaks (ICDD 43–1002) including (111), (200), (220) and (311) were observed on the XRD spectrum (Fig. 1b).

The synthesized CNPs were hydrophobic and could not disperse in water. Therefore, an alendronate-mediated surface modification method reported in our previous study was used to transfer the nanoparticles into water [29]. Three different ligands including SA, PEG600 and PEG2000 were conjugated to the surface of the CNPs (Fig. 1c). The CNPs coated by these ligands were named SA, PEG600 and PEG2000 respectively. The successful conjugation of these ligands onto the CNPs was confirmed by thermogravimetric analysis (TGA) (Fig. 1d). The morphologies of the surface modified nanoparticles were characterized by TEM. The results confirmed that the surface modification did not change the size and morphology of the nanoparticles (Fig. S1).

The ROS scavenging activity of the CNPs was evaluated by our previous method, where the O_2_^•−^ generated from riboflavin under light irradiation could be removed by the CNPs [29]. All of the constructed CNPs showed excellent ROS scavenging activity as they could realize over 90 % O_2_^•−^ inhibition ratio at very low concentration of 50 μg mL^−1^. The SA demonstrated the best ROS inhibition activity (Fig. 1e). The biocompatibility of the CNPs was evaluated by *in vitro* and *in vivo* experiments. The *in vitro* evaluation indicated that the CNPs did not inhibit the growth of the H9c2 (Fig. 1f–h) and HUVEC (Figs. S2a–c) cells even when the cerium reached very high concentration of 500 μM. The *in vivo* evaluation demonstrated that the CNPs did not impair the hepatic and renal function of the rats as the serum aspartate aminotransferase (AST), alanine aminotransferase (ALT), and serum creatinine (Cre) levels did not rise 24 h (Fig. 1i–k) and 28 d (Figs. S2d–f) after the injection of CNPs into the heart.

### 3.2 Therapeutic effects of CNPs on MIRI

**Fig. 2:**
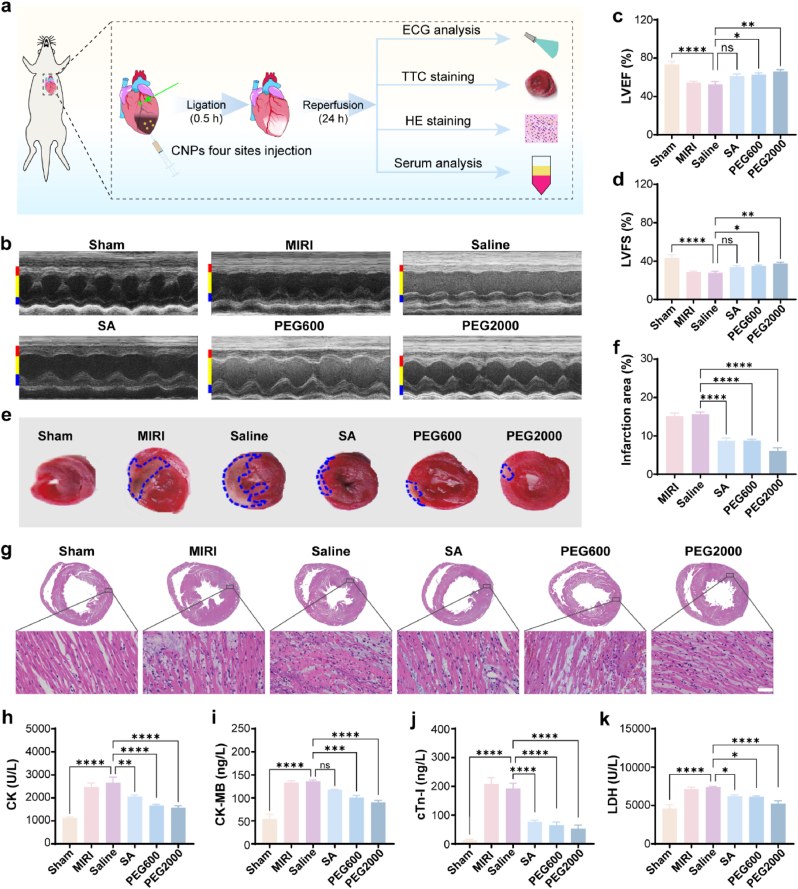
Therapeutic effects of CNPs on MIRI. (a) Scheme for the fabrication of the MIRI model and the pathway of the CNPs administration. (b) M-mode of echocardiography. The red, yellow, and blue ranges represent the left ventricular anterior wall, left ventricular cavity, and left ventricular posterior wall respectively (n = 6). (c–d) Quantitative LVEF and LVFS based on the ECG analysis (n = 6–7). (e) Myocardial infarction area test based on TTC staining. (f) Quantification of infarct size percentage based on the TTC staining. (Infarction area% = infarcted area/total area × 100 %, n = 6). (g) Representative HE staining panoramic images of cross-sections of the hearts (Scale bar = 50 μm). (h–k) Levels of the serum myocardial enzymes including CK, CK-MB, cTn-I and LDH (n = 6). Data were presented as mean ± SEM. ∗*p* < 0.05, ∗∗*p* < 0.01, ∗∗∗*p* < 0.001, ∗∗∗∗*p* < 0.0001, ns not significant.

The rat MIRI model was established through the ligation of the left anterior descending coronary artery for 30 min followed by the release of the ligation to allow the blood reperfusion. Once the ligation was released, the CNPs were injected into the anterior wall of the left ventricle by a four-point administration method. Echocardiography (ECG) analysis, 2,3,5-triphenyl-2H-tetrazolium chloride (TTC) staining, the heart tissue hematoxylin-eosin (HE) staining and serum analysis were performed 24 h after the injection of CNPs (Fig. 2a). The left ventricular M-mode ECG images revealed that the contraction and relaxation of the left ventricular anterior and posterior walls were severely attenuated in the MIRI group and saline group, especially the left ventricular anterior wall. Besides, the left ventricular pumping function parameters including the left ventricular ejection fraction (LVEF) and the left ventricular fractional shortening (LVFS) for the MIRI group and saline group were obviously lower than the sham group. These results confirmed the successful establishment of the MIRI model. Interestingly, in the SA group, PEG600 group and PEG2000 group, the systolic and diastolic capacity of the left ventricular myocardium was significantly improved compared with those in MIRI and saline groups, especially in the PEG2000 group (Fig. 2b). Meanwhile, the LVEF and LVFS in the SA group, PEG600 group and PEG2000 group were significantly improved, especially in the PEG2000 group (Fig. 2c and d). These results implied that the administration of CNPs during MIRI could reduce the acute injury of left ventricular wall and the ejection function of the left ventricle was therefore preserved.

The myocardial infarction area of the rats was determined by TTC staining. Large areas of myocardial infarctions were observed in the rats from MIRI group and saline group. However, in the SA group, PEG600 group and PEG2000 group, the sizes of myocardial infarctions were smaller than those in the MIRI group and saline group (Fig. 2e and f). On the other hand, it could be seen in the HE staining images that the ischemia-reperfusion resulted in the relax of the cardiac muscle fibers in the rats from MIRI group and saline group. Also, serious inflammatory infiltration, edema and nucleus broken were observed in the injured cardiac tissue in these two groups. While, the administration of CNPs, especially PEG2000, significantly attenuated the symptoms mentioned above (Fig. 2g). The serum myocardial enzyme analysis results indicated that the ischemia-reperfusion resulted in the raise of the creatine kinase (CK), creatine kinase isoenzyme (CK-MB), cardiac troponin I (cTn-I) and lactic dehydrogenase (LDH) levels as these parameters in the rats of MIRI group and saline group were obviously higher than that of the sham group. While, the administration of CNPs, especially PEG2000, could obviously decrease the expression of these enzymes (Fig. 2h–k). It could be concluded based on these observations that CNPs could effectively decrease the myocardial infarction and inhibit the serum myocardial enzymes.

In general, the CNPs featured with higher ROS scavenging activity could generate better therapeutic effects. This principle was verified by oxygen glucose deprivation (OGD) injury model of H9c2 cells, which indicated that the SA exhibited the best protecting effect on the cells (Fig. S3a). However, the *in vivo* results demonstrated that for most of the evaluated parameters, the PEG2000 showed the best therapeutic effects. The reason could be ascribed to the aggregation of SA under a physiological solution which hampered the diffusion of the nanoparticles to the tissues around the injection sites. To confirm it, three of CNPs stayed in the heart tissue was quantified by ICP-AES 24 h after injection. The results supported our hypothesis that the SA demonstrated the highest cerium concentration in the heart followed by PEG600 and PEG2000 (Fig. S3b).

### 3.3 CNPs inhibited cardiomyocyte apoptosis in ischemia-reperfusion region

**Fig. 3:**
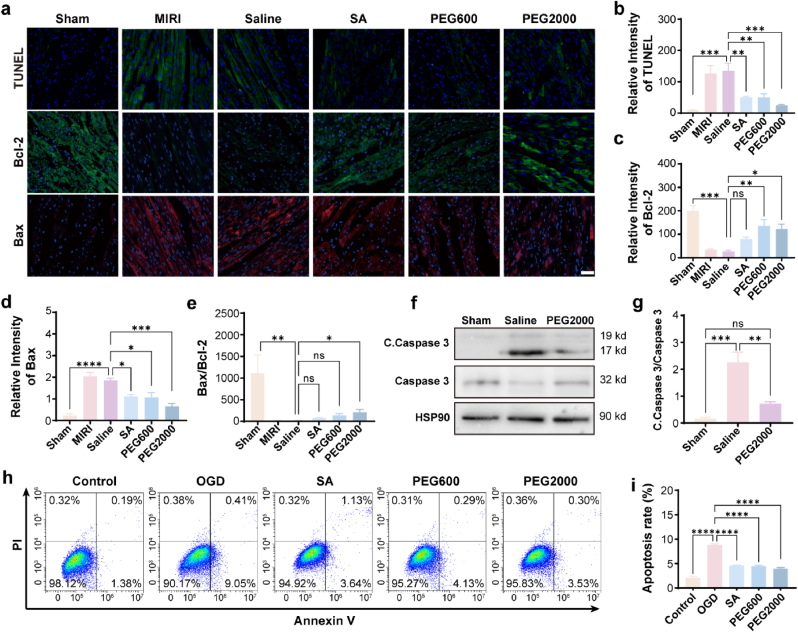
CNPs inhibited cardiomyocyte apoptosis in ischemia-reperfusion region. (a) Microscope images of the TUNEL staining, Bcl-2 staining and Bax staining (Scale bar = 50 μm). (b) Relative TUNEL positive areas in the heart tissues. (c) Relative Bcl-2 expression in the heart tissue. (d) Relative Bax expression in the heart tissue. (e) Bax/Bcl-2 ratio based on the data shown in c) and d) (n = 3). (f) Expression of Cleaved Caspase-3 (C. Caspase 3) and Caspase 3 in the heart tissue detected by western blot technology. (g) Quantified C. Caspase 3/Caspase 3 based on grayscale value (n = 3). (h–i) H9c2 cell apoptosis determined by Annexin-V staining and flow cytometry analysis (n = 4). Data were presented as mean ± SEM. ∗*p* < 0.05, ∗∗*p* < 0.01, ∗∗∗*p* < 0.001, ∗∗∗∗*p* < 0.0001, ns not significant.

Apoptosis of myocardial cells plays an important role in MIRI [30]^.^ Therefore, the inhibition of cardiomyocyte apoptosis must be a critical pathway for CNPs to attenuate the MIRI. To confirm it, *in vivo* and *in vitro* experiments were performed to probe the effects of CNPs on the apoptosis of cardiomyocytes. For the *in vivo* evaluation, the cardiac tissues were collected 2 h after reperfusion for TdT-mediated dUTP Nick-End Labeling (TUNEL) staining. The results indicated that almost no fluorescence was observed in the sham group indicating minimum apoptotic cells in the tissue, while strong fluorescence was observed in the tissues from MIRI group and saline group indicating the presence of quite a few apoptotic cells in the tissues. As expected, the fluorescence intensity in the tissues from three of the CNPs groups were much lower than that of the MIRI group and saline group (Fig. 3a and b). These observations confirmed that CNPs could effectively protect the cardiomyocytes from reperfusion induced apoptosis. To further confirm this conclusion, the apoptotic indicators including Bcl-2 and Bax were tested by immunofluorescence staining. It should be mentioned that the expression of Bcl-2 is negatively correlated with the apoptosis, i.e. the higher expression of Bcl-2 means the lower apoptotic cells [31]. In contrast, the expression of Bax is positively correlated with the apoptosis, i.e. the higher expression of Bax means the higher apoptotic cells [32]. It could be seen in the microscope images and the quantification data that the presence of CNPs resulted in the upregulation of Bcl-2 and downregulation of Bax (Fig. 3a, c-e). The quantification of apoptosis activation indicator, Cleaved-Caspase 3/Caspase 3, showed that the expression of Cleaved-Caspase 3 (C. Caspase 3) increased in the saline group, but significantly declined in the PEG2000 group (Fig. 3f and g). For the *in vitro* evaluation, the OGD cells were treated by CNPs and stained by Annexin-V for flow cytometry analysis. The percentages of the apoptotic cells including early and late apoptosis for the control group, OGD group, SA group, PEG600 group and PEG2000 group were 1.57 %, 9.46 %, 4.77 %, 4.42 % and 3.83 % respectively (Fig. 3h and i). Both of the *in vitro* and *in vivo* results reached the same conclusion that CNPs could effectively protect cardiomyocytes from apoptosis under ischemia and hypoxia conditions.

### 3.4 CNPs attenuated MIRI through the modulation of mitochondrial dynamics

**Fig. 4:**
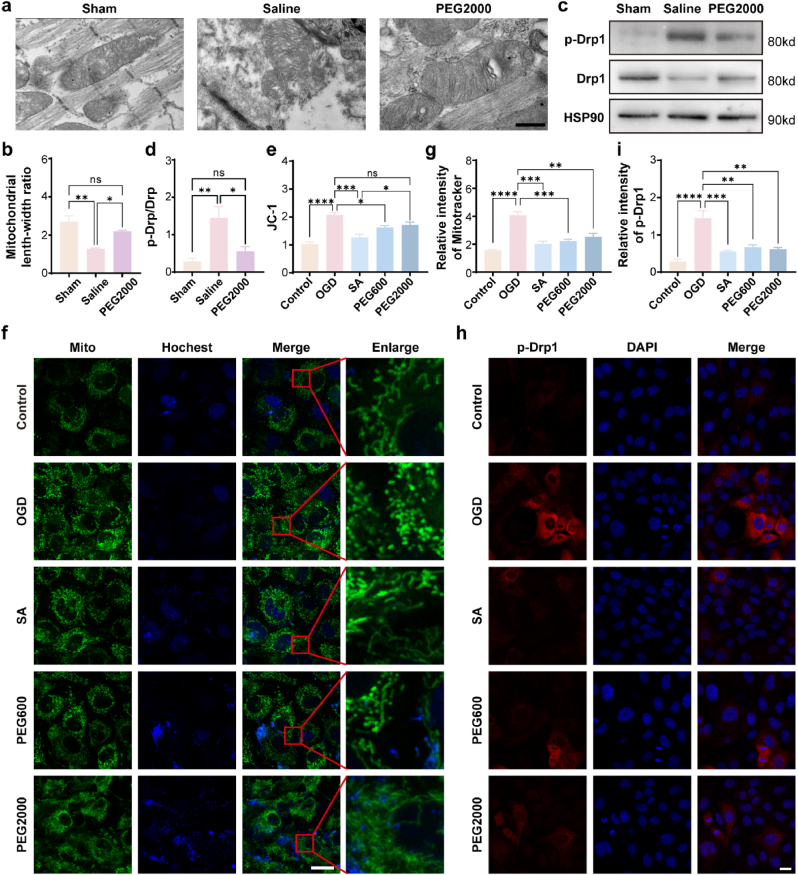
CNPs attenuated MIRI through the modulation of mitochondrial dynamics. (a) TEM images of mitochondria (Scale bar = 500 nm). (b) Quantified mitochondrial length-with ratio. (c–d) Expression of Drp1 and p-Drp1 quantified by WB technology. (e) Mitochondrial membrane potential detected by JC-1 fluorescent probe. (f) Mitochondrial morphology imaged by green fluorescent dye Mitotracker (Scale bar = 200 μm). (g) Quantified fluorescence intensity of Mitotracker. (h) Microscope images of the expression of p-Drp1 determined by immunofluorescence staining (Scale bar = 20 μm). (i) Quantified relative expression of p-Drp1 based on the fluorescent intensity. Data were presented as mean ± SEM. ∗*p* < 0.05, ∗∗*p* < 0.01, ∗∗∗*p* < 0.001, ∗∗∗∗*p* < 0.0001, ns not significant.

As mentioned above, mitochondrial dysfunction plays a critical role in the development of MIRI. We therefore believed that the protection of mitochondrial dynamics might be an important pathway for CNPs to protect the heart tissue from MIRI. To prove this hypothesis, *in vivo* and *in vitro* experiments were performed to probe the effects of CNPs on the dynamics of the mitochondria. For the *in vivo* evaluation, the morphologies of the mitochondria in the heart tissues were observed by TEM. Since PEG2000 exhibited the best effects in protecting the heart tissue from MIRI, this CNPs was selected to perform the experiment. The results indicated that the mitochondria of the Sham-operated group demonstrated tubular or rod-like morphology and almost no fragment was observed. In contrast, the mitochondria of the MIRI rat treated by saline showed swelling morphology and many fragments were observed in the cell. As expected, the administration of CNPs to the MIRI rat could effectively preserve the structure of the mitochondria as their morphologies were very close to the sham-operated rat (Fig. 4a). The quantification of the mitochondrial aspect ratio also reached the same conclusion (Fig. 4b). To further confirm the capability of CNPs in protecting the dynamics of the mitochondria, the expression of Drp1 was evaluated since this protein plays a central role in determining the fission and fragmentation of mitochondria. The activity of Drp1 is modulated by posttranslational modifications including phosphorylation, ubiquitination, sumoylation, S-nitrosylation, etc. [21] Within these, phosphorylation is of great importance in determining the activity of Drp1. Therefore, both of the p-Drp1 and Drp1 were quantified by western blot technology. The results indicated that the p-Drp1/Drp1 ratio in the heart tissue from the MIRI rat treated by saline was obviously higher than that from the sham rat. While, the administration of CNPs to the MIRI rat could effectively decrease the p-Drp1/Drp1 ratio (Fig. 4c and d).

For the *in vitro* evaluation, the OGD injury model was used to probe the effects of CNPs on the dynamics of mitochondria. The mitochondrial membrane potential was detected by JC-1 fluorescent probe [33]. Compared with the control group, the OGD cells showed obvious increase of mitochondrial membrane potential. As expected, the addition of SA, PEG600 and PEG2000 resulted in the recovery of mitochondrial membrane potential in the OGD cells (Fig. 4e). The mitochondria were labeled by green fluorescent dye Mitotracker. The confocal laser scanning microscope images revealed that the mitochondria in the control group exhibited tubular or rod-like morphology and few mitochondrial fragments were observed. However, a lot of mitochondrial fragments were detected in the OGD group. The addition of SA, PEG600 and PEG2000 to the OGD cells at the beginning of oxygen-glucose deprivation, which resulted in the restoration of the mitochondrial morphology (Fig. 4f and g). The *in vitro* immunofluorescence staining results indicated that the addition of CNPs could significantly decrease the expression of p-Drp1 (Fig. 4h and i). These observations were in accordance with the results of the *in vivo* experiments. Taking together of the *in vivo* and *in vitro* results, we have generated strong evidence that CNPs could reduce excessive mitochondrial fission under ischemia and hypoxia conditions by regulating mitochondrial dynamics.

### 3.5 CNPs reduce ROS levels in cardiomyocytes under IRI conditions both *in vitro* and *in vivo*

**Fig. 5:**
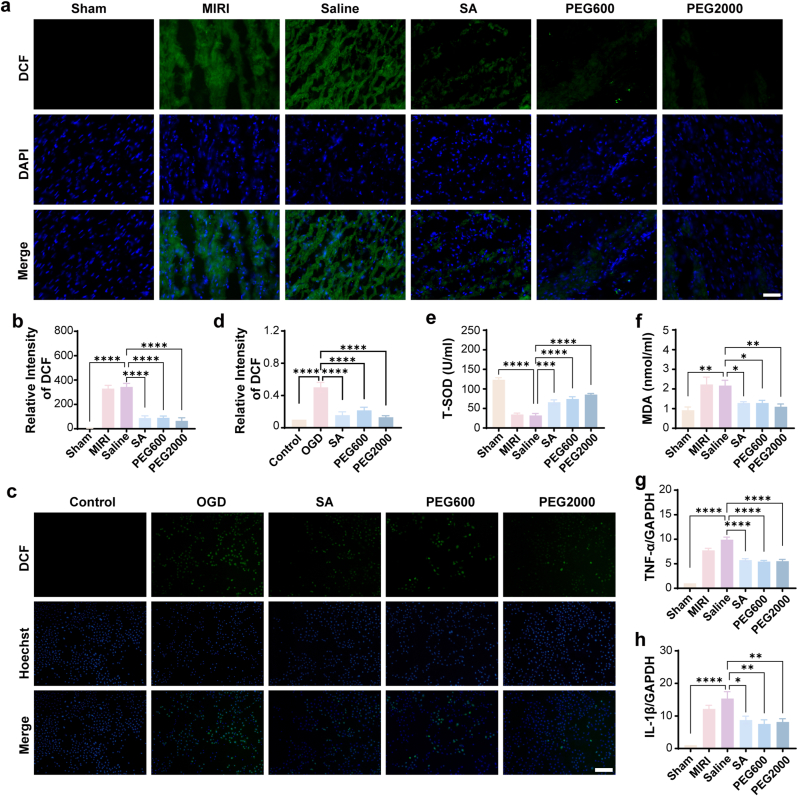
Pathway that the CNPs passed through to modulate the mitochondrial dynamics. (a) Microscope images of ROS level in the heart tissue detected by DCFH-DA staining (Scale bar = 50 μm). (b) Quantified relative DCF intensity in the heart tissue. (c) Microscope images of ROS level in OGD H9c2 cells detected by DCFH-DA staining (Scale bar = 50 μm). (d) Quantified relative DCF intensity in OGD H9c2 cells. (e) T-SOD concentration in the heart tissue. (f) MDA concentration in the heart tissue. (g–h) Relative expression of TNF-α and IL-1β. Data were presented as mean ± SEM. ∗*p* < 0.05, ∗∗*p* < 0.01, ∗∗∗*p* < 0.001, ∗∗∗∗*p* < 0.0001.

As mentioned above, ROS plays a central role in the pathogenesis of MIRI, and excess ROS production affects mitochondrial dynamics, ultimately impacting the metabolism and function of both mitochondria and the entire cell [34,35]. Meanwhile CNPs featured with strong ROS scavenge capability. Therefore, it was reasonable to believe that the modulation of ROS in the heart tissue was a key point of CNPs protection of the heart from ischemia-reperfusion injury. To prove this hypothesis, the ROS level in the heart tissue was evaluated by 2′,7′-dichlorofluorescein diacetate (DCFH-DA) staining 6 h after the reperfusion. The heart tissue from control group exhibited very low ROS level as almost no green fluorescence was detected in the tissue. However, in the MIRI group, strong DCF fluorescence was recorded due to the high ROS level in the heart tissue. As expected, the administration of CNPs could obviously decrease the ROS level (Fig. 5a and b). The feasibility of CNPs in the elimination of ROS was confirmed by the *in vitro* experiment performed on OGD H9c2 cells, which indicated that the intracellular ROS level was decreased in the presence of CNPs (Fig. 5c and d). The modulation of ROS level by CNPs was further characterized by the detection of total superoxide dismutase (T-SOD) and malondialdehyde (MDA) in the heart tissue. The results showed that CNPs promoted the expression of SOD and decreased the generation of MDA (Fig. 5e and f). By the way, the inflammation was inhibited by the CNPs as the expression of the two typical inflammatory factors in the cardiac tissue were obviously downregulated (Fig. 5g and h).

### 3.6 CNPs improved the long-term prognosis of MIRI rat

**Fig. 6:**
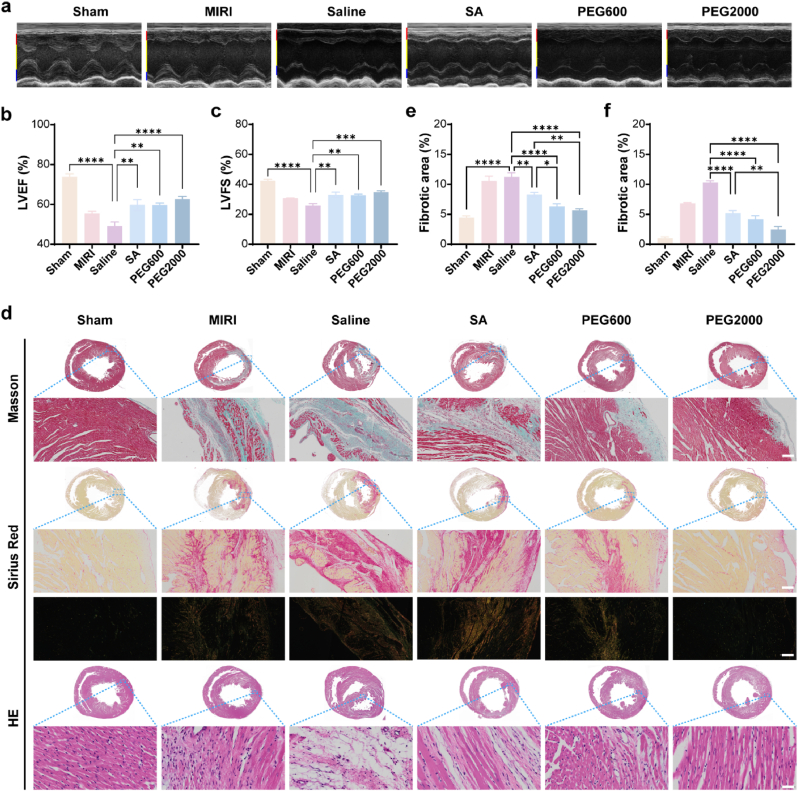
CNPs improved the long-term prognosis of MIRI rat. (a) M-mode of echocardiography. Red, yellow and blue ranges represented the left ventricular anterior wall, left ventricular cavity and left ventricular posterior wall respectively. (b–c) Quantified LVEF and LVFS based on the echocardiography analysis (n = 6). (d) Representative Masson, Sirius Red and HE staining images of heart cross sections (Scale bars for Masson and Sirius Red images were 200 μm, and scale bar for HE was 50 μm) (n = 6). Under ordinary optical microscope, collagen fibers were red. Under polarized light, type I collagen fibers were orange and type III collagen fibers were green. (e–f) Relative fibrotic areas quantified based on Masson staining and Sirius Red staining. Data were presented as mean ± SEM. ∗*p* < 0.05, ∗∗*p* < 0.01, ∗∗∗*p* < 0.001, ∗∗∗∗*p* < 0.0001.

The long-term prognosis i.e. the recovery of cardiac function was evaluated 28 days after the CNPs treatment. It could be seen from the M-mode echocardiography images that the left ventricular free wall motions in the MIRI group and the saline group were significantly impaired compared with the sham operation group. However, in the CNPs treatment groups, especially in the PEG2000 group, the left ventricular free wall motion was obviously improved (Fig. 6a). Echocardiographic data showed that the LVEF% and LVFS% of rats in CNPs treatment groups were significantly improved compared with those in the MIRI group and the saline group (Fig. 6b and c). It could be concluded based on these observations that CNPs could effectively protect the left ventricular systolic and diastolic function as well as the left ventricular ejection function 28 days after MIRI.

The cardiac function is highly related to the fibrosis of the heart tissue during MIRI. We therefore evaluated the collagen fibers in the heart tissue of the MIRI rats administered with CNPs 28 days after the treatment. The Masson's trichrome staining and Sirius red staining results indicated that significant heart tissue fibrosis was observed in the MIRI group and saline group. In contrast, the myocardial fibrosis was obviously inhibited in the MIRI rats treated by CNPs. The PEG2000 treated rats showed the lowest fibrosis within three of the CNPs groups (Fig. 6d–f). The pathologic results of HE staining confirmed the long-term protective effect of CNPs against MIRI (Fig. 6d). Based on these results, we believed that the CNPs reduced the ischemia-reperfusion injury during the acute stage, and thereby reduced the lateral myocardial fibrosis which was beneficial to the recovery of the cardiac function.

## 4 Conclusion

In this study, three surface coated CNPs, named SA, PEG600 and PEG2000 respectively, were synthesized and their potentials in the attenuation of MIRI were evaluated. Both of the short-term (24 h) and long-term (28 d) results indicated that the CNPs could effectively protect the structure and function of the heart tissue during MIRI. The PEG2000 showed the best therapeutic effect probably due to the better stability compared with the SA and PEG600 under physiological environment, which enabled the homogenization diffusion of the nanoparticles into the heart tissue around the injection site. The mechanism study revealed that the modulation of the over accumulated ROS triggered the lateral cascade reaction was the major reason for CNPs to attenuate the MIRI. Specifically, the elimination of ROS inhibited the oxidative stress in the cardiac tissue and reduced the phosphorylation of Drp1. Thus, excessive mitochondrial division was alleviated, ultimately reducing myocardial cell apoptosis during MIRI. The acute injury to the left ventricular wall caused by the MIRI was therefore attenuated and the ejection function of the left ventricle was preserved. These findings indicated that the antioxidant therapy based on the inorganic nanoparticles is a potential way for the treatment of MIRI.

## CRediT authorship contribution statement

**Ying Sun:** Writing – original draft, Methodology, Investigation, Data curation, Conceptualization. **Jiabao Xu:** Writing – original draft, Validation, Software, Project administration. **Ling Zou:** Software, Project administration, Methodology. **Yan Tan:** Resources, Investigation. **Jie Li:** Visualization, Validation. **Haoran Xin:** Supervision, Formal analysis. **Yanli Guo:** Data curation, Conceptualization. **Weikai Kong:** Methodology, Investigation. **Dingyuan Tian:** Writing – original draft. **Xinyu Bao:** Investigation. **Xiaoqin Wan:** Methodology. **Xiaoxu Li:** Methodology. **Zhihui Zhang:** Resources, Funding acquisition. **Xiaochao Yang:** Writing – review & editing, Supervision, Funding acquisition, Conceptualization. **Fang Deng:** Writing – review & editing, Supervision, Funding acquisition.

## Ethical approval

All animal surgical operations were performed in accordance with the Guidelines for Care and Use of Laboratory Animals of Army Medical University. Protocols were approved by the Animal Ethics Committee of Army Medical University (AMUWEC20228030).

## Declaration of competing interest

The authors declare that they have no known competing financial interests or personal relationships that could have appeared to influence the work reported in this paper.

## Data Availability

Data will be made available on request.
