# Supplementary material for: Ceria nanoparticles alleviate myocardial ischemia-reperfusion injury by inhibiting cardiomyocyte apoptosis via alleviating ROS mediated excessive mitochondrial fission
Source: Mater Today Bio. 2025 Apr 17;32:101770. doi: 10.1016/j.mtbio.2025.101770 (PMC12033917; doi:10.1016/j.mtbio.2025.101770)
Supplement: Multimedia component 1 [file mmc1.docx]

Supplementary Material

**Ceria nanoparticles alleviate myocardial ischemia-reperfusion injury by inhibiting cardiomyocyte apoptosis via alleviating ROS mediated excessive mitochondrial fission**

Ying Sun^a, bc, 1^, Jiabao Xu^a, c, 1^, Ling Zou^c, 1^, Yan Tan^a, d, e^, Jie Li^b, f^, Haoran Xin^b, f^, Yanli Guo^g^, Weikai Kong^h^, Dingyuan Tian^b, f^, Xinyu Bao^a, b, f^, Xiaoqin Wan^a, b, f^, Xiaoxu Li^d, e^, Zhihui Zhang^b, f, *^, Xiaochao Yang^c, **^, Fang Deng^a, de, ***^

^a^ Department of Pathophysiology, College of High Altitude Military Medicine, Army Medical University, Chongqing, 400038, China

^b^ Department of Cardiovascular Medicine, Center for Circadian Metabolism and Cardiovascular Disease, Southwest Hospital, Army Medical University, Chongqing, 400038, China

^c^ School of Biomedical Engineering and Medical Imaging, Army Medical University, Chongqing, 400038, China

^d^ Key Laboratory of Extreme Environmental Medicine, Ministry of Education of China, Chongqing, 400038, China

^e^ Key Laboratory of High Altitude Medicine, PLA, Chongqing, 400038, China

^f^ Key Laboratory of Geriatric Cardiovascular and Cerebrovascular Disease, Ministry of Education of China, Chongqing, 400038, China

^g^ Department of Ultrasound, Southwest Hospital, Army Medical University, Chongqing, 400038, China

^h^ Institute of Pathology and Southwest Cancer Center, Southwest Hospital, Army Medical University, Chongqing, 400038, China

*, **, ***Corresponding author.

^1^These authors contributed equally to this study.


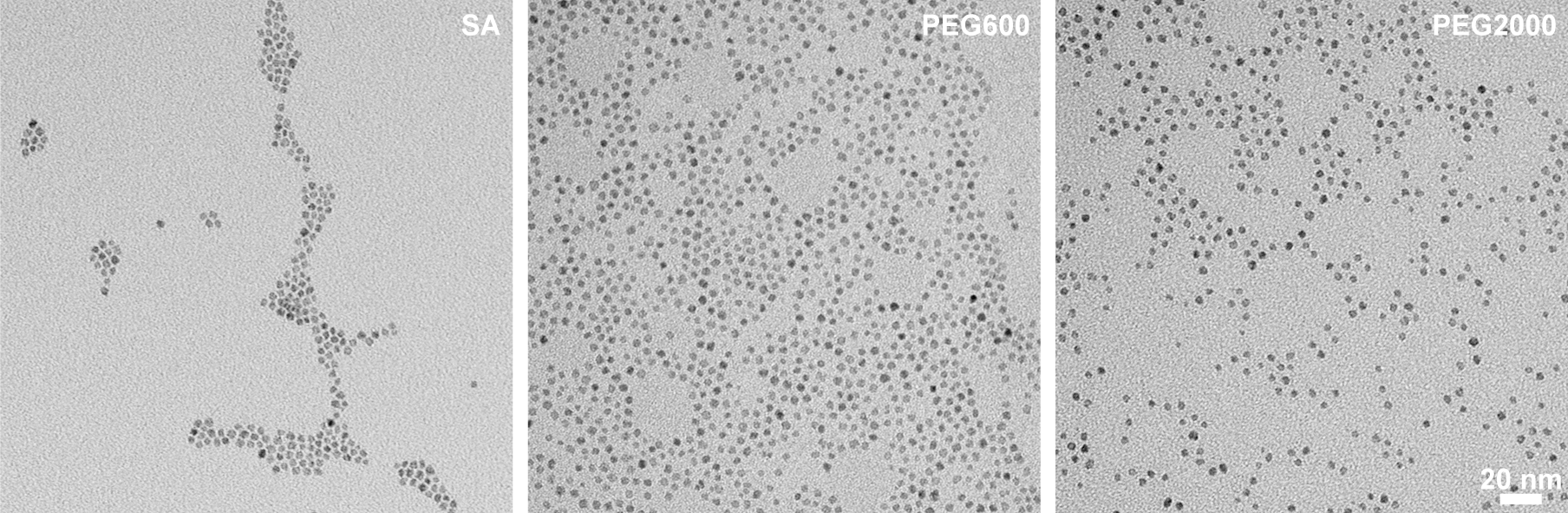


**Figure S1.** TEM images of ceria nanoparticles coated by different ligands.


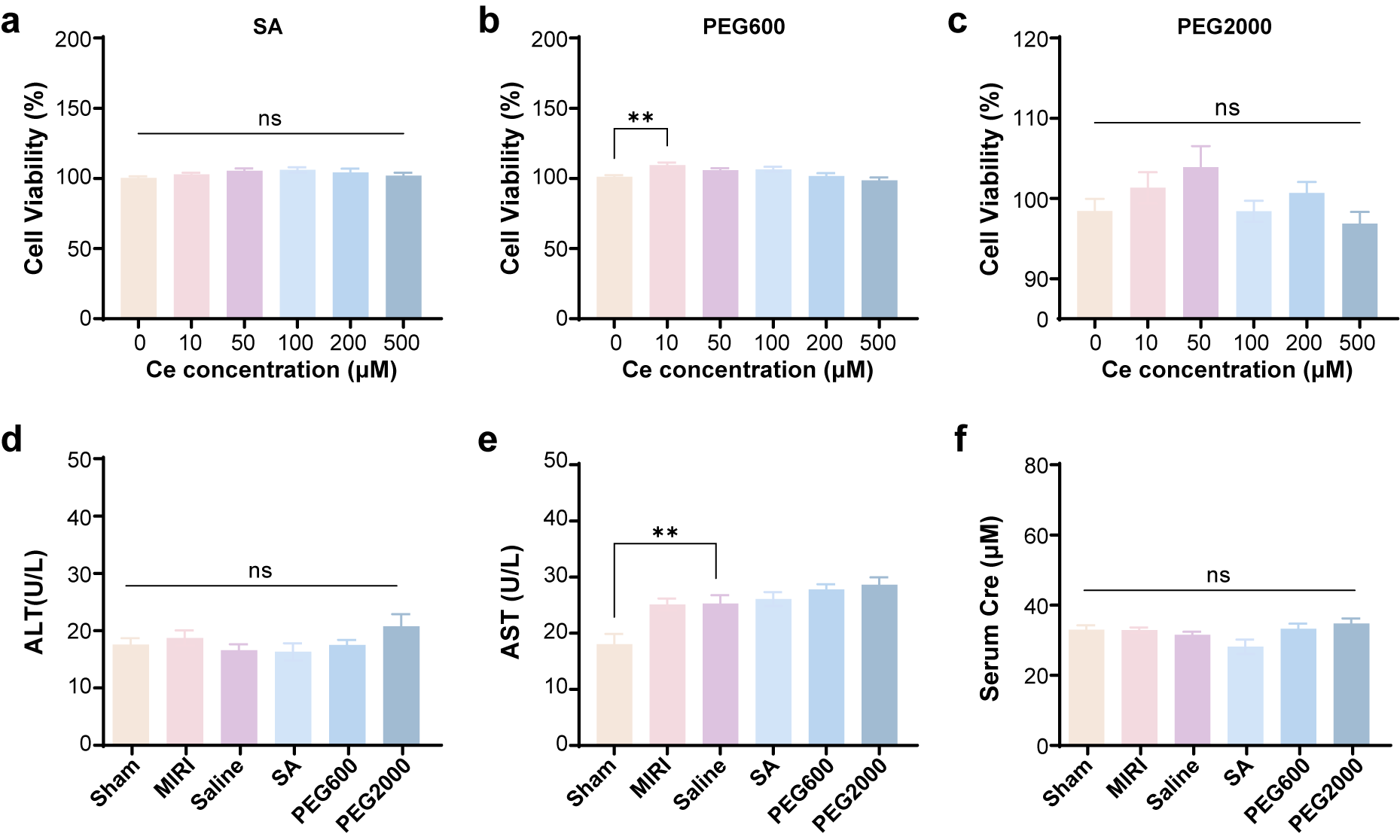


**Figure S2.** Biocompatibility of CNPs. (a-c) Cell viability of HUVEC of the surface coated CNPs measured by CCK-8 (n = 6). (i-k) Serum ALT, AST and Cre levels of the rats 28 d after the injection of CNPs (n = 6). Data were presented as mean ± SEM. ** *p* < 0.01, ns not significant.


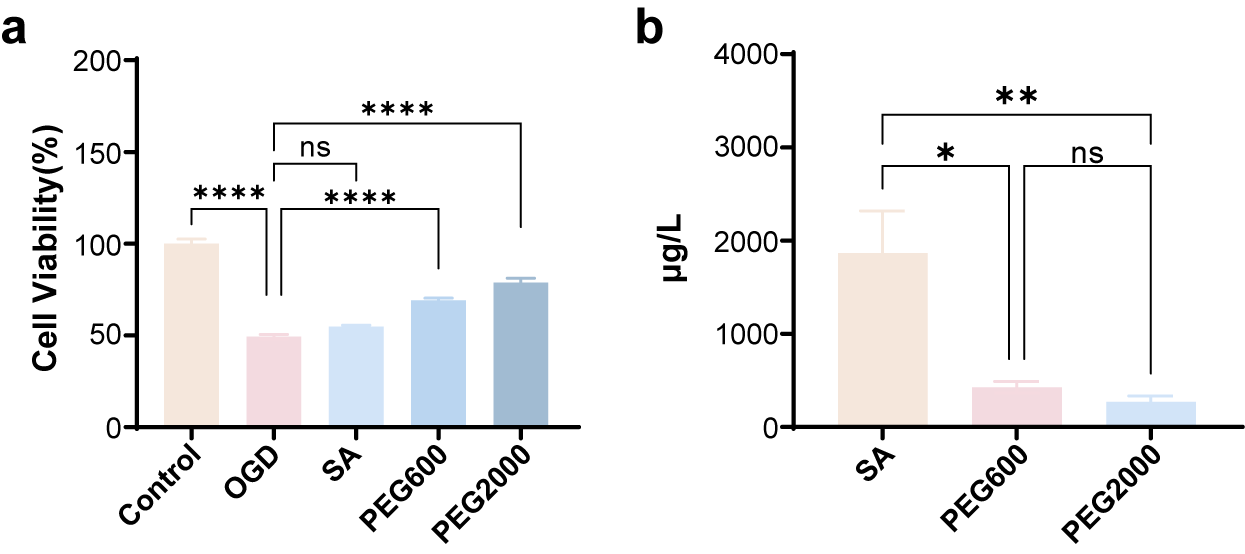


**Figure S3.** (a) H9c2 cell viability detected by CCK-8 after OGD injury (n = 6). (b) CNPs stayed in the heart tissue was quantified by ICP-AES 24 h after injection (n = 4). Data were presented as mean ± SEM. * *p* < 0.05, ** *p* < 0.01, **** *p* < 0.0001, ns not significant.

**Table S1.** Base Sequences of Relevant Genes for RT-PCR.

| mRNA | primer pairs (5′ → 3′) |  |
| --- | --- | --- |
| GAPDH | forward ACAGCAACAGGGTGGTGGAC reverse TTTGAGGGTGCAGCGAACTT |  |
|  |  |  |
| TNF-α | forward ATGGGCTCCCTCTCATCAGT reverse GCTTGGTGGTTTGCTACGAC |  |
|  |  |  |
| IL-1β | forward CCCAACTGGTACATCAGCACCTCTC reverse CCTGGGGAAGGCATTAGGAATAGTG |  |
|  |  |  |
